# Utilization of sexual and reproductive health services among construction worker women in southern Ethiopia

**DOI:** 10.1186/s12905-024-03042-x

**Published:** 2024-03-26

**Authors:** Tadele Dana Darebo, Zewudu Birhanu, Mihretu Alemayehu, Behailu Balcha, Adisu Worku, Desalegn Dawit Assele, Mark Spigt

**Affiliations:** 1 Department of Reproductive Health and Nutrition, School of Public Health, College of Health Sciences and Medicine, Wolaita Sodo University, Wolaita Sodo, Ethiopia; 2 2Department of public health, College of Health Sciences and Medicine, Wolaita Sodo University, Wolaita Sodo, Ethiopia; 3 Wolaita Zone Health Department, Wolaita Sodo, Ethiopia; 4 Department of Public Health, College of Medicine and Health Sciences, Hawassa University, Hawassa, Ethiopia; 5 Research Institute CAPHRI, Department of Family Medicine, Maastricht University, Maastricht, Netherlands; 6 General Practice Research Unit, Department of Community Medicine, UiT the Arctic University of Norway, Tromsø, Norway

**Keywords:** SRH services, Construction worker women, VDTS, Southern Ethiopia

## Abstract

**Background:**

In Ethiopia, the utilization of sexual and reproductive health services (SRH) is alarmingly inadequate, leading to higher rates of maternal and newborn mortality. Disparities in accessing sexual and reproductive health (SRH) services exist among different population groups, with construction worker women at a higher risk of experiencing such issues. We investigated the utilization of sexual and reproductive health services and associated factors among construction worker women in Southern Ethiopia.

**Method:**

We conducted a cross-sectional study among construction worker women (15–49) in Southern Ethiopia from July 1st to July 30th, 2021. The participants were selected randomly using venue-day-time sampling (VDTS). The data were collected by a pretested structured questionnaire using an open data kit (ODK) and exported to Statistical Package for Social Sciences (SPSS) version 25 for analysis. Binary logistic regression analysis was conducted to identify factors associated with sexual and reproductive health service utilization. An adjusted odds ratio with 95% CI was used and statistical significance was declared at *p*-value < 0.05.

**Results:**

The study revealed that 54.4% of women of reproductive age had used at least one sexual and reproductive health service in the past year. About 66.7% of women experienced sexual harassment at work, with sex discrimination (86.9%) and sexist hostility (57.9%) being the most common. Aged over 20 years, married women, living with husbands, friends, and boyfriends, within 30 min of health facilities, and having a favorable attitude were significantly associated with SRH service utilization.

**Conclusion:**

Nearly half of construction workers in southern Ethiopia are not using sexual and reproductive health services, indicating a concerning lack of access to such services. Over two-thirds of women experience sexual harassment in construction site. Therefore, to ensure universal access to SRH services, it is essential to design a new approach including outreach programs specifically tailored to reach such vulnerable groups.

## Introduction

Sexual and reproductive health encompasses the complete well-being of individuals, encompassing all aspects of sexuality and the reproductive system. It goes beyond specific reproductive health issues and concerns [1]. This comprehensive approach to sexual and reproductive health and rights ensures that individuals’ service needs are met throughout their reproductive lives [2]. However, 4.3 billion people worldwide lack access to essential interventions like prenatal, postnatal, contraceptive, and abortion care, despite primary healthcare units providing most of these services [3].

The burden is disproportionately high in low- and middle-income countries [4]; a staggering 214 million women of reproductive age lack access to modern contraception [5]. The lack of access to sexual and reproductive healthcare has increased unintended pregnancies and unsafe abortions [6]. Adolescent pregnancies are more prevalent in East Africa (21.5%), Northern Africa (9.2%), and Sub-Saharan Africa (19.3%) compared to Northern Africa [7]. Although abortion rates vary, developing regions experience 36 abortions per 1,000 women aged 15–44 annually, compared to 27 in industrialized regions [6].

Violence against women in the workplace is a significant public health issue [8]. The Fair Wear Foundation found that 75% of workers experienced verbal, physical, and psychological violence, while 60% of female garment workers experienced sexual harassment in factories [9]. Female factory workers in China, Bangladesh, and migrant workers in Vietnam are more susceptible to adverse sexual and reproductive health outcomes and sexual harassment [10–12]. Although the sexual risk behaviors were highly prevalent among factory workers. Factory workers in China exhibit high sexual risk behaviors, including condomless sex (23.6%), multiple sex partners (11.5%), and commercial sex (8.4%) [13].

Despite these challenges, they face barriers to accessing and utilizing services. Only 35% of female factory workers reported using sexual and reproductive health services [10], and only 41.3% of women in Shenzhen, China, used condoms during their sexual debut [13]. Women at work may face various sexual and reproductive health-related issues due to the nature of their work [14, 15]. Despite their higher-risk sexual and reproductive health issues, their utilization of these services has not been emphasized. There is a lack of information on the utilization rate and associated factors among construction worker women aged 15–49 in Ethiopia, including the study area. The study aims to assess the utilization of sexual and reproductive health services and its associated factors among construction worker women aged 15–49 in southern Ethiopia.

## Methods and materials

### Study area, period, and design

A cross-sectional study was conducted among construction worker women (15–49) from July 1st to July 30th, 2021, at the Wolaita Sodo Town. Wolaita Sodo town is the capital city of Wolaita Zone, which is 330 km from Addis Ababa (Ethiopian capital) and 270 km from Hawassa, the regional capital. The city was divided into four sub-cities, each of which contained 14 kebeles. There was one referral and teaching hospital, three government-owned health centers, and fourteen health posts, as well as four privately-owned hospitals, 54 clinics, and one non-governmental hospital.

### Population

All reproductive-age women attending construction work at Wolaita Sodo town construction sites were the source population, and all reproductive-age women (15–49 years) construction workers at randomly selected construction sites during the study period were the study population.

### Sample size determination

The sample size was calculated by single population formula by Epi info software version 7.2 considering the following assumptions: the anticipated proportion of 50% workers who received SRH service, a 95% confidence level, a 5% margin of error, and added 10% non-response. The calculated sample size for this study was 423.

### Sampling procedure

Reproductive-age women who attended daily labor work at construction sites were included in the sample using venue-day-time sampling (VDTS). Using a systematic method known as “venue-day-time sampling,” all construction sites in Sodo Town are identified and mapped, and then the average eligible groups present for daily work in each site are uniformly counted. Next, eligibility and participation preferences are determined. Finally, locations for the study were selected. When there is no obvious formal structure to apply the standard probability sampling approaches, such as when recruiting study participants in unusual areas like the street, among migrants, or other hidden populations, this sampling technique is advised [16, 17].

To determine the total daytime units and working conditions, some daily laborers at construction sites, the owner of the building, municipality administrative staff, site engineers, and community members were interviewed before the data collection. This led to the identity of 70 construction sites and using a standardized enumeration formula, it was found that there were on average 9 women of reproductive age working at a construction site each day (7 was the minimum and 20 was the maximum number).

Subsequently, 21 sites were randomly selected from the sample frame created during the formative assessment, and all study participants were carefully chosen from these 21 venues. The probability of selecting study units was based on the total number of venues and the proportion of women of reproductive age working as laborers at each construction site in Sodo Town. Weekly updates of the venues were implemented to ensure accurate sampling [18, 19], Weekly updates of the venues were implemented to ensure accurate sampling.

### Data collection tools, methods, and personnel

Pre-tested structured questionnaires in the English language were developed from other studies on SRH utilization of women of reproductive age in Ethiopia and elsewhere [20, 21]. The questionnaires include information on sociodemographic factors, health system-related factors, knowledge and attitudes towards SRH, and utilization of sexual and reproductive health services. Five diploma nurses with experience in data collection conducted face-to-face interviews using ODK while being supervised by two bachelor’s degree holder public health officers.

### Data quality management

To ensure accuracy, the data collection tool was translated from English to Wolaitigna by experts in that language. A pre-test was conducted on 5% of the sample size [22] who had worked in Bodit town construction sites. Data collectors and the supervisor were trained two days for consistency before the pretest, and the method of data collection, interview technique, and content of the questionnaire. The data collector always asked the participants if they had previously participated in a comparable study before commencing the interview to avoid reputation The data collected were checked for completeness and inconsistencies before analysis.

### Data analysis

Data were collected by ODK and then exported into Statistical Package for Social Sciences (SPSS) version 25. Descriptive statistics such as frequencies, proportions, and numerical summary measures were used to describe the data. Binary logistic regression analysis was conducted to identify factors associated with the utilization of SHR. Variables with a *p*-value less than 0.25 in bivariable analyses were a candidate for multivariable analysis. An adjusted odds ratio (AOR) with a 95% confidence interval was reported. Variables having a *p*-value less than 0.05 were considered statistically significant. Multicollinearity was checked among independent variables, which indicates no multicollinearity. The goodness-of-fit was checked using the Hosmer-Lemeshow test.

### Study variables

Dependent Variable (Sexual and reproductive health services utilization).

Independent Variable:

- Socio-demographic characteristics (age, educational status, Marital status, Family size, Occupation, and living arrangement).
- Health delivery system determinants (Distance, Continence of working hours, exempted services, and waiting time).
- Individual and behavioral related (Knowledge, attitude, and multiple sexual partners).

## Operational definitions

### SRH services utilization

This was determined by asking participants if they had used at least one or more SRH service components (family planning, STI service, VCT, abortion service, antenatal care, delivery, postnatal care, maternal waiting area) within the last twelve months, and the response was dichotomous (yes or no). Additionally, the positive response was further validated with each question on the type of SRH services utilized. A positive (“yes”) response to at least one SRH service was regarded as service utilization [22].

### Knowledge toward sexual and reproductive health service

Eight awareness questions with Yes and No alternate answers were used to measure respondents’ understanding of sexual and reproductive health services. Since the total score can vary from 0 to 8, responders with scores of 5.74 or above were deemed knowledgeable [23].

### Attitude towards SRH services

A respondent was deemed to have a positive attitude toward SRH services if their mean score on five attitude-related items on a 1–5 Likert scale was greater than or equal to 15.07 [22].

### Waiting time

It was calculated as the number of minutes a client had to wait after arriving at a health facility before receiving sexual and reproductive health treatments. If it lost less than or equal to 30 min, it was coded 1; otherwise, it was marked 0 [24].

### Distance to nearby health facility

Measured from the report of women on the walking hours to the health facilities. This was coded 1 if women reported the walking hours = < 30 min to reach the nearby health facility; otherwise, it was coded 0 if it was greater than 30 min [25].

### Exempted service

It refers to a service that is offered without charge to all clients who are women of reproductive age to achieve public health objectives when the service provision falls short of expectations because externalities are present [26].

### Multiple sexual partners

Women having more than one sexual partner in the last twelve months [27].

## Results

### Socio-demographic characteristics

A total of 412 participants with a response rate of 97.4% who worked as laborers at the construction site were included in the study. The mean age of the women was 22.8 ± 5.221 years, and nearly half (47.1%) of them were between the ages of 20 and 24. The majority of women (66%) had completed their elementary education, and 251 (or 60.9%) were unmarried (Table 1).

**Table 1:** Socio-demographic characteristics among daily worker women (15–49) years at Sodo Town construction venues, Wolaita Sodo, August 2021 (*n* = 412)

Variable	Frequency (n)	Percent (%)
**Age groups (in years)**		
15–19 years	163	39.6%
20–24 years	194	47.1%
> 24 years	55	13.3%
**Marital status**		
Single	251	60.9%
Married	157	38.1%
Widowed/Divorced	3	0.94%
**Educational status**		
No formal education	39	9.5%
Primary school	272	66%
Secondary school	87	21.1%
College and above	14	3.4%
**Living arrangements**		
Living with parents	156	37.9%
Living with husbands	77	18.7%
Living with friends	136	33%
Others(boyfriends)	43	10.4%

### Health delivery system characteristics

Among the total respondents, 319 (77.4%) have a service-delivery facility in their neighborhood, 276 (67%) of the women said it takes less than or equal to 30 min to walk to the closest health facility, and 171 (41.5%) said the service point was open during their visit (Table 2).

**Table 2:** Health delivery system characteristics among daily worker women (15–49) years at Sodo Town construction venues, Wolaita Sodo, August 2021 (*n* = 412)

Variable	Frequency (n)	Percent (%)
**The presence of a facility in the living area**		
Yes	319	77.4%
No	93	22.6%
**Waiting time for service**		
Less than or equal to 30 min	204	49.5%
Greater than 30 min	208	50.5%
**Distance to the nearest health facility**		
Less than equal to 30 min in walking time	276	67%
Greater than 30 min in walking time	136	33%
**Convenient working time**		
Service points not closed through their visit time	171	41.5%
Service points closed through their visit time	241	58.5%

### Knowledge and attitude towards SRH services and related problems

Among the total respondents, 239/412 (58%) of the respondents who were women of reproductive age were aware of at least one service for sexual and reproductive health and the issues that surround it. Among the 58% of respondents who were aware of at least one SRH service and associated issue, 362/412 (87.1%) of them had heard of at least one SRH service; 304 (73.4%) were aware of how HIV and AIDS are transmitted; 315/412 (76.5%) were aware of family planning methods; 236/412 (57.3%) were aware of harmful traditional practices; 252 (61.2%) were aware of at least one MCH service; 215 Health professionals accounted for 61.7% of information sources, followed by radio 49%, peers 47.1%, and posters 21.8%. The majority of respondents, 233 (56.6%), had a positive opinion of the SRH services (Table 3).

**Table 3:** Knowledge and attitude towards SRH Services among daily worker women (15–49) years at Sodo town construction sites

Variable	Frequency (n)	Percentage (%)
Heard at least one SRH services		
Yes	362	87.9
No	50	12.1
Know the family planning methods		
Yes	315	76.5
No	97	23.5
Know the modes of HIV transmission		
Yes	304	73.8
No	108	26.2
Know the mode of transmission of STI		
Yes	215	52.2
No	197	47.8
Know the signs and symptoms of STI		
Yes	57	13.8
No	355	86.2
Know the modes of STI transmission		
Yes	190	46.2
No	222	53.8
Know the maternal and child health (MCH) services		
Yes	252	61.2
No	160	38.8
Know the harmful traditional practices		
Yes	236	57.3
No	176	42.7
Overall knowledge		
Knowledgeable	239	58.0
Not knowledgeable	173	42.0
**Attitudes toward SRH services**		
Favorable attitude	249	60.4%
Non favorable attitude	163	39.6%

### The experiences of sexual and reproductive health-related issues

**Fig. 1:**
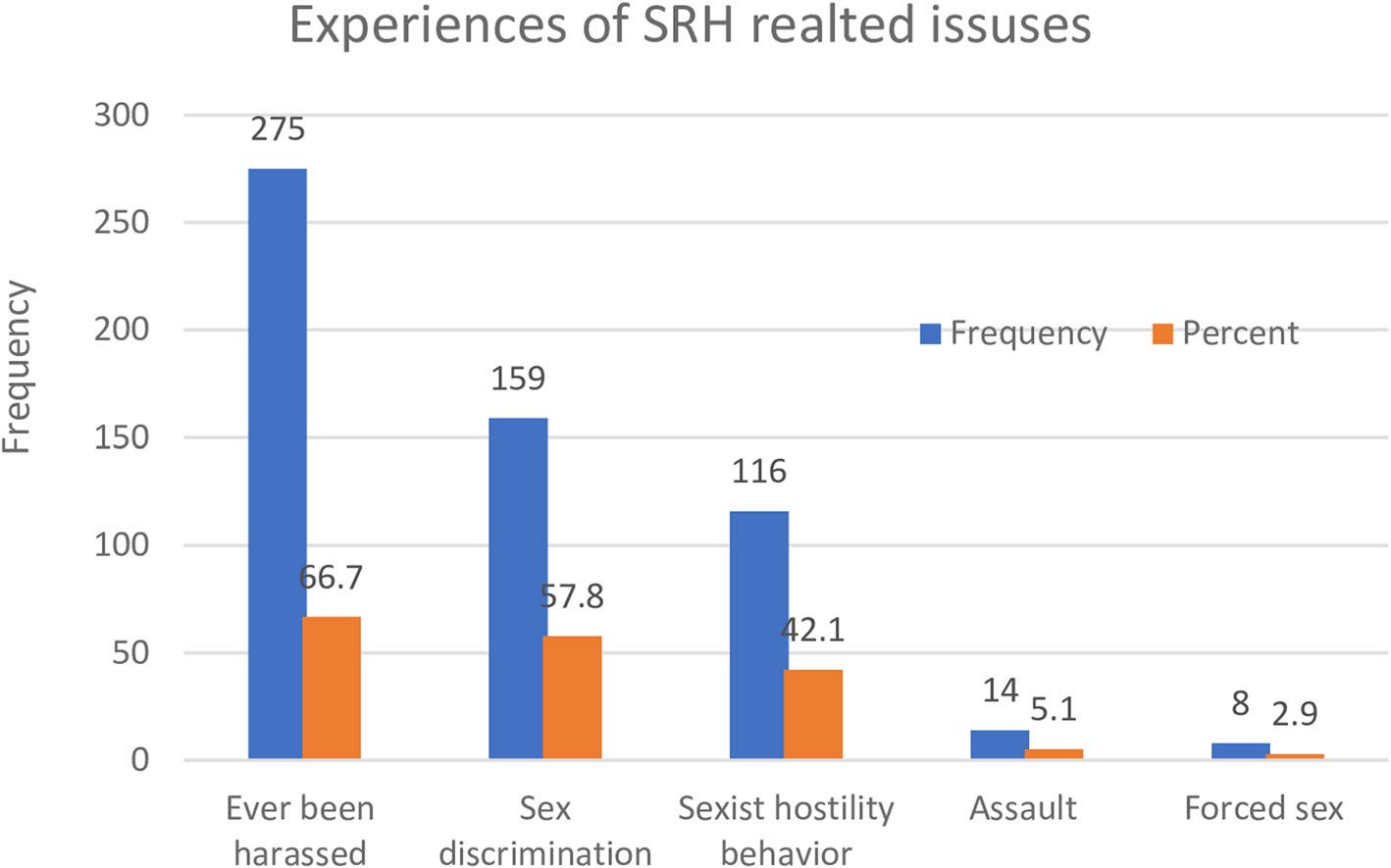
Reproductive-age women had experienced harassment at the construction site

Among the participants, 275 (66.7%) of the total respondents reported having experienced harassment on the job. Among the 275 individuals who reported being harassed (66.7%), 86.9%, 57.9%, 5.1%, and 2.8% reported having ever been the victim of sexism, violence, or forced sex, respectively. 11% of respondents had engaged in unprotected intercourse at some point in the previous year (Fig. 1).

### Utilization of SRH services

The study found that 224 (54.4%) of construction workers had used at least one SRH service in the last 12 months. The contraceptive methods were the most popular SRH service, used by 131 (31.8%) of participants. Forty-six (11.2%) participants used injectables, 10.4% preferred implants, 6.1% used oral pills, 3.9% used condoms, and 1% used intrauterine contraceptive devices. Furthermore, 34 (8.3%) women had been pregnant within the past year; of them, 61.8% used antenatal care services and 85.3% used postnatal care services (Table 4).

**Table 4:** The sexual reproductive health service utilization among daily worker women (15–49) years at the Sodo Town construction site

Characteristics	Frequency	Percentage
Utilized at least one SRH services		
Yes	224	54.4
No	188	45.6
SRH services utilized in the last 12 months (*n* = 224)		
Utilization of family planning	131	31.8
Types utilized		
Injectable	46	11.2
Implant	43	10.4
Oral pills	25	6.1
Condom	16	3.9
IUCD	4	1
Utilized STI treatment	33	8
VCT utilization		
Counseled for HIV test	104	25.2
Tested for HIV	106	25.7
Who knew the test result	93	22.6
History of pregnancy	34	8.3
Utilized safe abortion care	1	2.9
Utilized ANC service	21	61.8
Maternal waiting for home utilization	5	14
Utilized Post-natal care	29	85.3
Place of delivery		
Health facility	27	79.4
Others	6	18
Utilized SRH information	57	13.8

### Factors associated with the utilization of SRH service

In bivariate analysis, age, marital status, education, living arrangement, presence of facility, distance, working hour convenience, knowledge, and attitude were found to be statistically significant for SRH service utilization.

In multivariate logistic regression analysis, the age group of 20–24 years and above 20 years were 4 times more likely to utilize the SRH services than the age groups of 15–19 years (AOR = 4.09, 95%CI [2.24–7.45]) and 4 times more likely (AOR = 4.36, 95%CI [1.80-10.57]), respectively. Married women were 6 times more likely to utilize the SRH services than their counterparts (AOR = 6.01, 95%CI [3.28-11.0]). Respondents living with their husband were 6 times more likely to utilize the service (AOR = 5.93, 95%CI [2.63–13.36]), while those living with a friend were 4 times more likely (AOR = 4.26, 95%CI [2.22–8.18]), and those living with boyfriends were 4 times more likely (AOR = 4.26, 95%CI [1.72–10.57]), compared to those living with their parents. Participants living within a distance of less than or equal to 30 min of walking to reach nearby facilities were 4 times more likely to utilize the sexual and reproductive health services than their counterparts (AOR = 4.17, 95%CI [2.29–762]). Women who had a favorable attitude were 5 times more likely to utilize the SRH services than those who had a non-favorable attitude (AOR = 5.38, 95%CI [3.06–9.44]) (Table 5).

**Table 5:** Multivariable logistic regression analysis to identify factors associated with SRH services utilization among daily worker women (15–49) years at the construction site, Wolaita Sodo town, Ethiopia, August 2021

Variables	Utilization of SRH services		COR (95%CI)	AOR (95%CI)
	Yes (%)	No (%)		
**Age**				
15–19 years	59 (36.2%)	104 (63.8%)	1 (ref)	1 (ref)
20–24 years	128 (66%)	66 (34%)	3.41 (2.21–5.28)	4.1 (2.24–7.4)**
Above 24 years	37 (67.3%)	18(32.7%)	3.62 (1.89–6.92)	4.3 (1.80–10.5)*
**Marital status**				
Single	96 (38.2%)	155 (61.8%)	1 (ref)	1 (ref)
Married	128 (79.5%)	33 (20.5%)	6.26 (3.95–9.91)	6.0 (3.28-11.0)**
**Educational status**				
No formal education	10 (25.9%)	29 (74.4%)	1 (ref)	1 (ref)
Primary education	154 (56.6%)	118 (43.4%)	3.78 (1.77–8.07)	1.02 (0.35–2.92)
Secondary education	52 (59.8%)	35 (40.2%)	4.30 (1.86–9.94)	1.24 (0.39–3.92)
College and above	8 (57.1%)	6 (42.9%)	3.86 (1.07–13.90)	0.47 (0.08–2.74)
**Living arrangement**				
With parents	51 (32.7%)	105 (67.3%)	1 (ref)	1(ref)
With husband	50 (64.9%)	27 (35.1%)	3.81 (2.14–6.77)	5.93 (2.63–13.36)**
With friends	94 (69.1%)	42 (30.9%)	4.60 (2.81–7.55)	4.26 (2.22–8.18)**
With others (boyfriend)	29 (67.4%)	14 (32.6%)	4.26 (2.07–8.76)	4.26 (1.72–10.57)**
**The presence of a facility in the living area**				
No	31 (33.3%)	62 (66.7%)	1 (ref)	1 (ref)
Yes	193 (60.5%)	126 (39.5%)	3.06 (1.884–4.98)	1.23 (0.61–2.49)
**Distance**				
> 30 min of walking	35 (25.7%)	101 (74.3%)	1 (Ref)	1 (ref)
<= 30 min of walking	189 (68.5%)	87 (31.5%)	6.26 (3.95–9.93)	4.17 (2.29–7.62)**
**Working hour convenience**				
No	112 (46.5%)	129 (53.5%)	1 (ref)	1 (ref)
Yes	112 (65.5%)	59 (34.5%)	2.18 (1.45–3.27)	1.29 (0.74–2.266)
**Knowledge on SRH**				
Poor knowledge	77 (44.5%)	96 (55.5%)	1 (ref)	1 (ref)
Knowledgeable	147 (61.5%)	92 (38.5%)	1.99 (1.33–2.96)	1.29 (0.74–2.20)
**Attitude toward SRH services**				
Non favorable attitude	56 (34.4%)	107 (65.6%)	1	1 (ref)
Favorable attitude	168 (67.5%)	81 (32.5%)	3.96 (2.60–6.02)	5.38 (3.06–9.44)

*Significant at a *p*-value < 0.05 level and ** significant at a *p*-value < 0.001 level

*AOR* Adjusted odds ratio, *COR *Crud odds ratio, *CI *Confidence interval, *SRH *Sexual and reproductive health

## Discussion

The study aimed to assess the utilization of sexual and reproductive health services among women of reproductive age employed in construction work in Wolaita Sodo, southern Ethiopia. Due to the unique nature of this population group, there is limited existing literature specifically focused on sexual and reproductive health service utilization in similar settings. However, it is valuable to provide an overview of the findings in relation to studies conducted in other contexts on sexual and reproductive service utilization.

Violence against women is a significant human rights violation, with a high percentage of women reporting harassment, including verbal and physical abuse, sexual harassment, forced labor, assault, and rape [28]. This study revealed that 66.7% of women experienced sexual harassment at work, with sex discrimination and sexist hostility being the most prevalent issues. Sexual harassment is prevalent in the workplace, with approximately 60% of factory workers in India and Bangladesh experiencing verbal or physical abuse [9].

The study revealed that 54.4% of women of reproductive age had used at least one sexual and reproductive health service in the past year. This finding is higher compared to a study conducted in the rural district of the Sidama region, where only 37% of respondents had used SRH services [20]. The variation in utilization rates may be attributed to the study’s area and sociodemographic characteristics. Rural areas may have a greater awareness gap and longer distances to health facilities, limiting access to SRH services. However, the Sidama region has higher utilization of family planning services, possibly due to early marriage prevalence, resulting in more married women using contraceptive methods without hesitation [20].

The study found that women aged 20–24 were four times more likely to use SRH services compared to those aged 15–19. This finding is consistent with studies conducted in Addis Ababa, Western Ethiopia, and Eastern Africa, which demonstrated that youth aged 20–24 years were more likely to utilize SRH services compared to those aged 15–19 years [29–31]. As individuals age, they gain more decision-making autonomy and may engage in marital relationships, potentially contributing to the utilization of Social Support Housing (SRH) services.

Living arrangements significantly influence SRH service utilization, with women living with husbands or friends being five and four times more likely to use SRH services compared to those living with parents. This finding aligns with studies conducted in the West Arsi Zone of Oromia region and Addis Ababa, which indicated that individuals not living with their families and those who discussed SRH service issues with their friends or family were more likely to utilize SRH services [21, 29]. This indicates that women residing with their parents or relatives may encounter obstacles in accessing Social Security Health (SRH) services due to family influences and restrictions.

Marital status significantly influences SRH service utilization, with married women being six times more likely to use SRH services compared to unmarried or single women. This finding is consistent with studies conducted in Ghana, Nekemte, and Addis Ababa, which found that being unmarried or not engaged in any marital relationship decreased the likelihood of seeking SRH services [29, 32–34]. The cultural and social pressures may lead married women to feel more comfortable and willing to use sexual and reproductive health (SRH) services compared to unmarried women.

The proximity to health facilities significantly influences the utilization of SRH services, with women living within a 30-minute distance being four times more likely to use these services. This finding is consistent with studies conducted in Kenya, Ethiopia, and Egypt, which highlighted the impact of proximity on seeking medical attention and access to care [35–37]. Limited access to services due to long distances acts as a barrier to the utilization of sexual and reproductive health services.

The study found that respondents’ attitudes significantly influenced their decision to use SRH services, with positive attitudes being five times more likely for women to use these services. This finding is consistent with studies conducted in South Ari and other countries, which indicated that individuals with positive attitudes towards SRH services were more likely to utilize them [38–41]. Positive attitudes can increase the demand for SRH services by encouraging individuals to seek medical attention and actively seek information about these services.

The respondents’ knowledge of sexual and reproductive health, including HIV prevention methods, is a crucial indicator of reproductive health coverage [42]. The study revealed that 26.2% of participants had insufficient knowledge about HIV transmission, and 42% had insufficient knowledge about SRH. Access to sexual and reproductive health (SRH) services is linked to increased awareness of SRH, although more than half of respondents have inadequate SRH knowledge in Italy [43] and Lebanon [44]. This underscores the need for targeted awareness and education campaigns to enhance understanding of HIV transmission and sexual health, thereby enabling individuals to make informed decisions [45].

### Strengths and limitations of the study

To the best of the researcher’s knowledge, this study could be the first to measure SRH utilization among construction worker women in Ethiopia. Another strength could be applying venue-daytime sampling (VDTS) techniques, which are specially designed for such studies. The limitations could be, that the cross-sectional design used in this study cannot establish a temporal relationship between the dependent and independent variables. Additionally, obtaining honest responses from women, especially those under the age of 18 may have been challenging due to the sensitive nature of the topics.

## Conclusion

The study revealed that almost half of the women of reproductive age working at a Wolaita Sodo town construction site do not access SRH services, highlighting a significant disparity. The study found that age, marital status, and place of residence are significant demographic factors that influence women’s access to substance misuse (SRH) services, indicating the need for targeted interventions. We suggest that catchment health facilities and other stakeholders should enhance the provision of sexual and reproductive health (SRH) services on construction sites. Strengthening outreach services is crucial to bridge the gap and improve utilization among construction worker women. Addressing barriers like age, marital status, and residence can lead to tailored interventions for equitable access.

In conclusion, this study underscores the importance of addressing the poor utilization of SRH services among women of reproductive age working at the construction site in Wolaita Sodo town. By focusing on improving outreach services and considering the specific factors influencing service utilization, significant progress can be made in enhancing the SRH outcomes and well-being of this vulnerable population.

## Data Availability

The data will be available from the corresponding author upon justifiable requests.

## Abbreviations

AOR: Adjusted odds ratio
EDHS: Ethiopian demographic health survey
MCH: Mother and child health
ODK: Open data collection kit
SNNPR: Southern nations nationality, and people region
SRH: Sexual and reproductive health
SDG: Sustainable development goal
VCT: Voluntary counseling and testing
VDTS: Venue-day-time sampling
